# Barriers and facilitators of intuitive eating in postmenopausal women: A qualitative study

**DOI:** 10.1177/20551029231157515

**Published:** 2023-02-18

**Authors:** Jeanne Vorlet, Isabelle Carrard

**Affiliations:** 1 Metabolic Center, 386507 Intercantonal Hopital De La Broye Site Payerne, Switzerland; 2 Department of Nutrition and Dietetics, 128870 HES-SO Genève, Switzerland

**Keywords:** Intuitive eating, body image, barriers, facilitators, implementation, women, menopause, overweight, obesity

## Abstract

This qualitative descriptive research explored barriers and facilitators of the intuitive eating (IE) implementation process, as experienced by six postmenopausal women classified as ‘overweight’ or ‘obese’. The data was analysed using deductive and inductive thematic analysis and six themes were identified. IE implementation included developing scepticism about weight loss diets, dealing with hunger and satiety cues, making nutritious food choices for one’s body, struggling with emotional eating, learning to accept one’s body and challenging weight stigma and sociocultural norms of beauty and thinness. IE allowed women to develop a more peaceful relationship with their diet, and was accepted as a welcome alternative to dieting.

## Introduction

The prevalence of overweight and obesity, as defined by the World Health Organization (WHO, 2020),^1^ is increasing (OECD, 2019). Western society has promoted a culture where thinness is considered a beauty ideal (Polivy and Herman, 2004). Persons classified as ‘overweight’ or ‘obese’ are encouraged to reach a medically standardised “healthy weight” through lifestyle changes (Rodgers, 2016). According to the sociocultural model by Rodgers’ (2016), people who do not meet the standards conveyed by this “healthy weight” are more likely to develop body shape and food concerns. Similarly, the internalisation of the sociocultural ideal of thinness, and comparisons with this unrealistic ideal can lead individuals, and especially women, to develop a negative body image (Van Den Berg et al., 2002). Women classified as ‘overweight’ are also more likely than women within the ‘healthy’ body mass index (BMI, weight in kg/height in square meter) range to suffer from body dissatisfaction (Weinberger et al., 2016). Perceived overweight is associated with a desire for weight loss, and an increased likelihood of engaging in weight loss attempts (Haynes et al., 2018). Unfortunately, restrictive weight loss diets often have disappointing results (Franz et al., 2007; Siahpush et al., 2015) and potentially undesirable consequences on health, such as the development of pathological eating behaviour (Polivy and Herman, 1985; Quick and Byrd-Bredbenner, 2012; Stice et al., 2017), long term weight gain (Siahpush et al., 2015), and increasing health problems with age (Krieger et al., 2006; Shapses and Riedt, 2006; Weinheimer et al., 2010).

**Figure 1.**
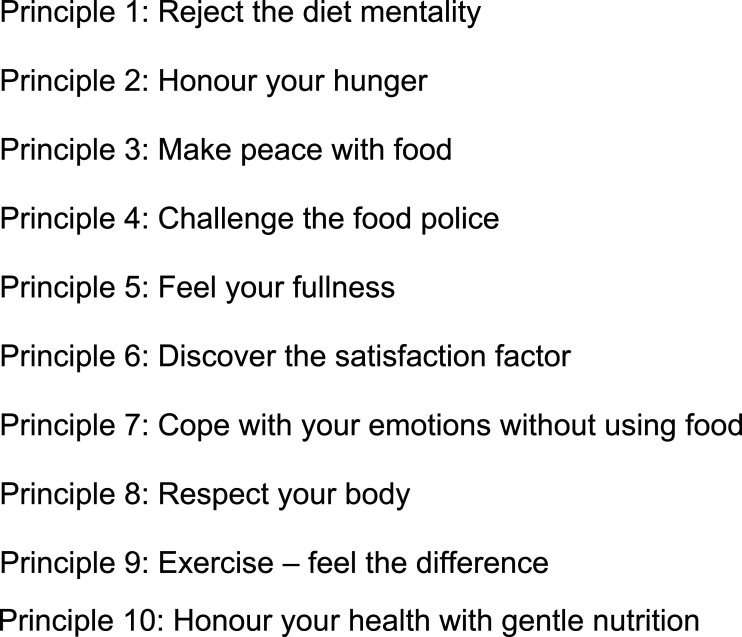
10 principles of intuitive eating by Tribole and Resch (2012).

**Figure 2.**
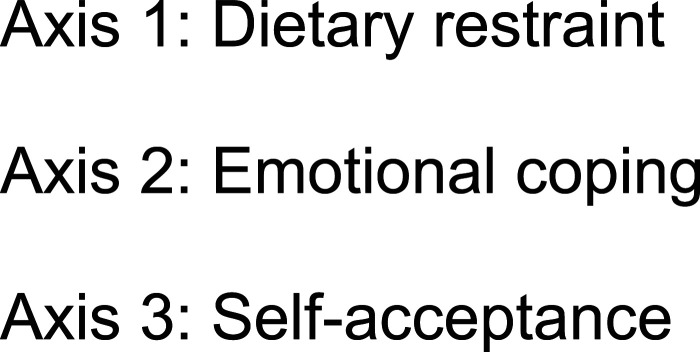
Three axes of the triaxial psycho-sensory approach of the GROS.

Intuitive eating (IE) emerged in the United States in the 1990s as a new treatment paradigm for obesity (Gast and Hawks, 1998). This approach promotes an eating pattern based on physiological hunger and satiety cues, and encourages a healthy relationship with food, physical activity and the body (Tribole and Resch, 2012). IE was developed by the American dieticians Tribole and Resch (2012), who proposed 10 principles, for learning or relearning to eat intuitively (Figure 1). This anti-diet approach is mainly known and applied in English-speaking countries. Tribole and Resch’s (2012) book “*Intuitive Eating: A revolutionary program that works*” was only recently translated into French, bringing knowledge of their 10 principles to a larger French-speaking audience. In parallel to this paradigm shift, the French Think Tank on Obesity and Overweight (Groupe de Réflexion sur l’Obésité et le Surpoids, GROS) has proposed a triaxial psycho-sensory approach (Figure 2) to supporting people suffering from overweight or obesity (Sweerts et al., 2020). This approach, which is comparable to the principles of IE, has since been widely applied in the French-speaking countries of Europe. The three axes advocated by the GROS focus on reducing dietary restraint, which is defined as the successful or unsuccessful intention to mentally control one’s diet in order to stabilise or lose weight (Herman and Polivy, 1975). Their approach also addresses internal food cues, emotional eating and the reinforcement of self-acceptance. This last concept aims to teach an acceptance of one’s body with its imperfections and to develop self-assertion and self-respect in a world that is stigmatising for persons classified as ‘overweight’ or ‘obese’ (Sweerts et al., 2020).

Whereas intuitive eating has attracted the interest of researchers, and the number of related studies is growing, the GROS triaxial approach has so far been investigated in only one pilot study (Sweerts et al., 2020). This pilot study showed no significant differences in weight, dietary restraint, emotional eating or intuitive eating scores in a 3-month GROS intervention as compared to a traditional diet. Due to the scarcity of research on GROS interventions, the present paper makes use of the scientific literature on IE. The two approaches align in their focus on reducing diet mentality and dietary restraint, enhancing hunger and satiety cues, reducing emotional eating, and developing gustative satisfaction through mindful eating. While the core principles of the two approaches are very similar, they differ in two themes. Tribole and Resch (2012) propose a chronological process ending with gentle nutrition as the 10th principle of IE, which is not present in the GROS approach. Secondly, the GROS proposes a third axis of self-acceptance, which isn’t a focus of the 10 IE principles. On the whole, the two approaches are highly complementary and were therefore both included in the present work.

The body of evidence linking IE and various health outcomes is growing. Cross-sectional studies have demonstrated a positive association between IE and better psychological well-being, such as self-esteem, self-compassion and life satisfaction, as well as a higher body appreciation (Bruce and Ricciardelli, 2016; Linardon et al., 2021). Overall, IE has been inversely related to eating disorder symptomatology (Babbott et al., 2022; Linardon and Mitchell, 2017; Tylka and Kroon Van Diest, 2013; Van Dyck et al., 2016), depression, anxiety and stress (Babbott et al., 2022). IE has also been negatively associated with body image concerns or dissatisfaction in different population samples (Linardon and Mitchell, 2017; Tylka and Kroon Van Diest, 2013; Van Dyck et al., 2016). More specifically, in a French population, IE was negatively associated with restraint eating, emotional eating and uncontrolled eating (Camilleri et al., 2015). Many cross-sectional studies have also demonstrated an inverse association between IE and BMI (Camilleri et al., 2015; Horwath et al., 2019; Tylka and Kroon Van Diest, 2013), and higher IE scores were associated with less weight cycling and with weight stability over time (Tylka et al., 2019; Van Dyke and Drinkwater, 2014). Nevertheless, IE implementation showed no significant weight loss in intervention studies (Fuentes Artiles et al., 2019). The effect of IE interventions on cardiovascular risk factors remains controversial to this day (Khasteganan et al., 2019). A longitudinal study in adolescents and young adults, however, found long-term benefits of an IE intervention. Both higher baseline IE scores and increases in IE scores from baseline to follow-up were found to be associated with lower odds of body dissatisfaction, and unhealthy or extreme weight control behaviours (Hazzard et al., 2021). A systematic review of 20 studies on interventions promoting IE, such as *Health at Every Size* (HAES) or mindful eating programs, found that IE interventions reduced food disinhibition and binge eating, and also led to a better response to hunger and satiety cues (Schaefer and Magnuson, 2014). Overall, this data is encouraging, and reinforces the interest in promoting IE instead of restrictive weight loss diets.

Nevertheless, IE can be difficult to implement when confronted with the societal pressure of the thinness ideal (Polivy and Herman, 2004), as well as the “healthy weight” norms (Rodgers, 2016). These health- and appearance-related pressures may weaken reliance on internal food cues and instead encourage eating according to external rules, which could explain lower IE scores among women with higher BMI (Augustus-Horvath and Tylka, 2011). Although the number of empirical studies on IE is growing (Beintner et al., 2019; Boucher et al., 2016; Burnette and Mazzeo, 2020; Cole and Horacek, 2010; Richards et al., 2017), few studies have investigated the implementation of, and adherence to, such an approach to date. Burnette and Mazzeo (2020) conducted a pilot study among college women that included an 8-week IE group or guided self-help (GSH) intervention. Both interventions promoting IE demonstrated feasibility, with 63% of participants completing the IE group intervention and 84% completing the GSH intervention (Burnette and Mazzeo, 2020). An analysis of a satisfaction questionnaire also demonstrated a high acceptability of the two interventions among the participants. Moderators and mediators of the treatment effect were also investigated (Burnette et al., 2022). Interoceptive awareness, self-compassion, body acceptance, and cognitive and behavioural flexibility were increased after the IE interventions, highlighting some of the mechanisms at play (Burnette et al., 2022). These results (Burnette et al., 2022; Burnette and Mazzeo, 2020) encourage future interventions promoting IE. Adherence is essential in improving health indicators and psychological well-being in the long term (Bradshaw et al., 2010). One way to help individuals adopt better health behaviours relies on identifying and understanding the barriers and facilitators to these behavioural changes (Michie et al., 2011). The present study, therefore, focused on the barriers and facilitators that could be encountered throughout the IE implementation process. This should provide clinicians accompanying patients through the implementation of IE with some understanding of factors affecting its implementation and indications of how to mitigating barriers or enhance facilitators.

Two qualitative studies have explored the barriers and facilitators of learning how to eat intuitively (Barraclough et al., 2019; Erhardt, 2021). One of the studies was conducted in New Zealand by Barraclough et al. (2019) among 11 women aged between 40 and 50 who were classified as ‘overweight’, and the second study was conducted by Erhardt (2021) among eight women aged 26–48 years in the United-Kingdom. IE seemed to be a natural behaviour that was lost during childhood and required time, effort and skills to reclaim (Barraclough et al., 2019). Several environmental and sociocultural barriers were identified, such as the dominant dieting culture (Erhardt, 2021), and the influence and judgment of others (Barraclough et al., 2019; Erhardt, 2021). Protecting and detaching oneself from others’ opinions, for example, was identified as a facilitator of IE (Barraclough et al., 2019; Erhardt, 2021). These two studies were conducted among young and premenopausal women. Although the body of evidence is growing, postmenopausal women are still poorly represented in IE studies (Bruce and Ricciardelli, 2016; Linardon et al., 2021). Elderly women, however, have shown lower IE-scores than younger women (Augustus-Horvath and Tylka, 2011). There is increasing evidence that body dissatisfaction persists with age (Cameron et al., 2019), and that a desire for thinness incited older women to follow weight loss diets (Carrard et al., 2018; Gagne et al., 2012), thus distancing them from the IE principles (Tribole and Resch, 2012). These women were as likely as younger women to develop eating disorders (Gagne et al., 2012) and suffer from the consequences related to these restrictive practices, such as nutritional deficiencies (ANSES, 2010), and loss of lean mass with an increased risk of sarcopenia (Weinheimer et al., 2010) or reduced bone density (Shapses and Riedt, 2006). It is essential to avoid restrictive practices and to promote a balanced diet in this population, and this is an important determinant of “*healthy aging*” (Atallah et al., 2018). Because menopause is often associated with physiological changes and weight gain (Karvonen-Gutierrez and Kim, 2016), it can be considered a period of vulnerability for many women to develop body dissatisfaction and eating disorders (Mangweth-Matzek et al., 2013). It is therefore important to understand the challenges of implementing IE in this population, for whom this approach could have many benefits. The present qualitative study explored how postmenopausal women classified as ‘overweight’ or ‘obese’ experience IE implementation in their daily lives. It focused on describing the barriers and facilitators encountered throughout this process.

## Methods

### Research design

This study involves qualitative research based on an interpretative paradigm with a descriptive approach according to Sandelowski (2000). From a naturalistic perspective, this approach aims to describe the phenomenon of interest by remaining close to the collected data (Sandelowski, 2000). The ontological position of such an approach is relativism, which views reality as subjective and variable from one person to another (Bradshaw et al., 2017). The descriptive approach was ideal in this study to obtain new knowledge directly from the concerned women. Thematic data analysis was used (Braun and Clarke, 2006).

This study was conducted in the French-speaking part of Switzerland in a Metabolic Centre specialising in the treatment of patients classified as overweight or obese. The IE approach, and more specifically the three axes of the GROS, are used in the various treatments proposed in this Metabolic Centre. The first author was a dietician in this Centre. The population attending this establishment comes mostly from an agricultural area (Department of Finance and External Relations, 2018; Statistical Office, 2013). To our knowledge, no study concerning IE has yet been conducted with a rural sample.

### Participants

Six women were recruited through convenience sampling within the Metabolic Centre. This corresponds to the lowest number of participants recommended by Braun and Clarke (2013), and is sufficient for a thematic analysis of the collected data. To reduce the risk of social desirability bias (Butori and Parguel, 2012) due to recruiting her own patients, the first author benefited from the support of dietician and psychotherapist colleagues, who selected the patients who met the inclusion criteria from their own patient pool. At this point, the selected participants received an information sheet and had to give their verbal consent to be contacted by the first author to discuss the study procedures. Participants were informed of the interviewer’s dietician title. Even though the first author communicated her researcher identity and her non-judgmental position to the participants, this could have influenced some of the responses in the interviews.

The inclusion criteria were the following: women; 55–65 years old; BMI ≥25 kg/m^2^; followed at the Metabolic Centre by a dietician according to the IE or GROS approach. The age limit of 55–65 years was chosen in order to recruit women who had completed the menopausal transition phase, a period of great physiological and hormonal changes which is often accompanied by constraining side effects (Harlow et al., 2012). It seems that women classified as ‘obese’, between 50 and 65 years of age are more affected by body dissatisfaction, by the desire for thinness, and by disinhibited eating than women past 65 years of age (Lewis and Cachelin, 2001). The chosen age category therefore captured the stories of women who might use weight loss behaviours to address body dissatisfaction. The exclusion criteria were post-bariatric surgery status, diagnosis of bulimia nervosa or binge eating disorder according to the DSM-5 (Diagnostic and Statistical Manual of Mental Disorders, fifth edition) (American Psychiatric Association, 2013), and Liraglutide treatment, because of their effect on women’s eating behaviour and body image (American Psychiatric Association, 2013; Ivezaj and Grilo, 2018; Pilitsi et al., 2019).

### Data collection and analysis

Data collection occurred through semi-structured individual interviews (Bradshaw et al., 2017; Sandelowski, 2000) by the first author between November 2020 and January 2021. The interviews lasted between 60 and 90 min, and took place via videoconference. The open-ended questions included in the interview guide (Annex 1) first addressed the participant’s experience of the IE implementation process by asking about the encountered barriers and facilitators. The second part addressed the participants' body weight concerns, body image acceptance and eating behaviour. The participants were also invited to reflect on the evolution of their eating behaviour and body image in recent years. The interview guide was pre-tested to ensure a good understanding of the questions with a person not involved in the project. The interviews were recorded by a voice recorder. The interviewer conducted this study as part of her Master’s degree in Health Sciences and was supervised by the second author. She had already acquired experience in qualitative research. The first author also had 4 years of experience in the treatment of overweight and obesity, as well as in the IE and the GROS approaches. Being convinced of the benefits of these two approaches, the first author had to reflect on her preconceptions, perspectives, and values throughout this project. Discussions held with the co-author and readings from the scientific literature regarding IE, body image and menopause helped her to acquire a critical point of view, and to step away from her position as a clinician. An audit trail recording the principal reflections and decisions made with the co-author was created and completed throughout data collection and analysis.

The recordings were transcribed orthographically into verbatim statements (Braun and Clarke, 2013). The transcripts were then analysed according to the six steps of Braun and Clarke’s (2006) thematic analysis. The first step involved an initial familiarisation with the transcripts and observation notes, allowing an initial search for patterns. For that matter, transcripts were read multiple times. Secondly, a semantic and complete coding was carried out using NVivo software. The analytical process was carried out at a descriptive level. The analysis of the data corpus was carried out deductively and inductively, as complementary approaches. A first list of deductive codes was created on the theoretical basis of the existing concepts of IE and body image (for example food cues, food rhythm, emotional eating, body appreciation, and body weight). As barriers and facilitators to the implementation of IE have been little studied so far, the inductive or data-driven approach allowed new codes to be identified from the collected data set (for example ambivalence about moving away from dieting, self-assertion, weight stigma) that had not been identified in the first theory-driven approach. An iterative approach was used for the data coding, involving a constant back-and-forth movement between the transcripts. In order to identify new codes, the first author started to complete the list of codes by analysing the first three interviews, before starting the coding again from the beginning, and then doing the same for the last three interviews. During this procedure, the first author coded all extracted data related to the research question and objectives. The third step consisted of analysing the codes and gathering them into broader themes. In the fourth step, after many exchanges between the first and second author, the themes most relevant to the research question were selected and revised. In Step Five, the themes were named and linked to the three axes of the GROS approach (Sweerts et al., 2020), and the 10 IE principles of Tribole and Resch (2012). Finally, the sixth step consisted of reporting the results and providing examples with verbatim statements from the participants (Braun and Clarke, 2006).

### Ethical procedures

The study protocol (*n*° 2020–01,896) was accepted by the local ethics committee. Each participant received an information sheet before recruitment by the first author, informing them about the study procedures and their right to withdraw from the research at any time. Written informed consent was signed by each participant before being interviewed. At the time of the study, each participant was in therapy with a dietician or psychotherapist from the Metabolic Centre. As the study subject may have been emotive for participants, they were invited to address any discomfort or distress arising from the interviews with their assigned therapists. To ensure anonymity, participants were assigned a coded name (P1, P2, P3, P4, P5, P6). The recordings were deleted after transcription. The participants’ personal data was guaranteed by coded storage on a computer at the Metabolic Centre only accessible by the first author. At the end of the study procedures, the transcriptions and the signed written informed consent forms were archived for a 10-year period at the university where the second author is working. It was not planned to use these data for future studies.

## Results

Six patients from the Metabolic Centre were interviewed. All had been in treatment with dieticians, psychologists, experts in adapted physical activity and specialised doctors. Table 1 presents their characteristics.

**Table 1.** Participant characteristics.

Participants	Age	IE experience
P 1	65	1 year
P 2	57	1.5 years
P 3	57	1.5 years
P 4	61	1 year
P 5	60	4–5 years
P 6	55	5 months

Note: IE = Intuitive Eating; P = participant.

The thematic analysis identified six themes that reflect aspects necessary for the IE implementation process as experienced by the participants. The barriers and facilitators of the IE implementation process are described for each theme (Table 2).

**Table 2.** Three axes of the psycho-sensorial approach of the GROS, six themes of the present study, 10 principles of intuitive eating by Tribole and Resch.

Three axes of the GROS	Six themes of the present study	Barriers and facilitators		10 principles of intuitive eating by Tribole and Resch
1^st^ axis Dietary restraint	1. Developing scepticism about weight loss diets	Barriers	Presence of significant weight concerns	Principle 1: Reject the diet mentality Principle 3: Make peace with food Principle 4: Challenge the food police
		Facilitators	Experiencing the drawbacks of weight loss diets	
	2. Dealing with hunger and satiety cues	Barriers	Hunger: Family obligations or schedules	Principle 2: Honour your hunger Principle 5: Feel your fullness
			Satiety: Self-indulgence in food, the influence of others	
		Facilitators	Hunger: Allowing flexibility in one’s eating rhythm, anticipating one’s need to eat	
			Satiety: Pleasant meal environment, self-assertion	
	3. Making nutritious food choices for one’s body	Barriers	Using a balanced diet in order to lose weight	Principle 6: Discover the satisfaction factor Principle 10: Honour your health with gentle nutrition
		Facilitators	Finding satisfaction in the consumption of nutritious and balanced foods, trusting one’s body to guide food choices	
2^nd^ axis Emotional coping	4. Struggling with emotional eating	Barriers	Needing to overeat to inhibit emotions, upsetting events, mood fluctuations	Principle 7: Cope with your emotions without using food
		Facilitators	Welcoming emotions and taking care of them, engaging in pleasant activities, perceiving emotionally charged situations differently, self-assertion	
3^rd^ axis Self-acceptance	5. Learning to accept one’s body	Barriers	Presence of significant weight concerns	Principle 8: Respect your body Principle 9: Exercise – feel the difference
		Facilitators	Rethinking weight loss expectations, taking care of one’s body	
	6. Challenging weight stigma and sociocultural norms of beauty and thinness	Barriers	Internalisation of sociocultural norms of beauty and thinness, weight stigmatisation	No principle
		Facilitators	Developing self-assertion, learning to listen to oneself, regaining self-confidence	

## Theme I: Developing scepticism about weight loss diets

Becoming sceptical about the misleading promises of weight loss diets would facilitate the acceptance of the anti-dieting IE approach and is therefore one necessary step of the IE implementation process. Experiencing the drawbacks of dieting facilitated the sceptical reflections around dieting and even helped some participants to move away from restrictive diets. However, the presence of significant weight concerns resulted in a desire to restrict eating in an attempt to lose weight, thus creating ambivalence about moving away from dieting behaviour.

All participants had experienced a history of dieting, food restrictions, and feelings of guilt when eating “forbidden” foods. Through their experiences, they realised the drawbacks of weight loss diets over the long term. Dieting was seen as restrictive and boring, as it offers a poor variety of allowed foods, and is socially complicated, expensive and time-consuming. Diets had a strong psychological effect, as illustrated by a participant (P6) who became “*aggressive*” and whose mood changed when dieting. Four participants experienced weight regain after weight loss: “*I managed to lose ten kilos during a diet but regained them straight after.*” (P6). Dietary restriction would also lead to frustration ending in food compensation.

“*When I forced myself not to eat certain foods, these foods would chase me, so they were things that I wanted to eat, and all of a sudden, I would lose control and I take the example of the chocolate bar […], instead of eating a small piece, I could eat the entire bar. […]. Because in a way, we'll go back to everything we haven't eaten lately*!” (P2)

In this case, learning to eat palatable foods without feeling guilty about it decreased food-associated frustration and resulting overconsumption. Experiencing these negative effects of dieting led the six participants to develop scepticism about weight loss diets, which in turn facilitated their acceptance of the anti-dieting IE approach. Some participants had decided not to engage in dieting behaviour anymore. “*I decided not to follow any diet. Because at some point, I realised ‘weight loss diets don’t work for me’ and I stopped following them*” (P5). Another woman exclaimed, “*tell people to not follow stupid diets because it makes you sick!*” (P4).

However, even though the participants were aware of the undesirable consequences of dietary restraint, several women struggled to move away from diet mentality. According to them, the desire to lose weight was one of their main reasons for engaging in dieting behaviour. P6 described falling back into restrictive eating or weight loss diets as soon as her weight increased. The need to lose weight still guided the behaviour of some participants: “*despite everything, I still restricted myself a little, because otherwise I can never achieve it [to lose weight]!*” (P3). Unresolved ambivalence could explain the difficulty encountered by several participants in moving away from weight loss diets. *“But how could I lose more [weight], without falling back into the strict diet cycle?”* (P2). The IE approach was, however, considered a valuable alternative to dieting, as P4 explained: “*we can also live better, without all these constraints*".

## Theme 2: Dealing with hunger and satiety cues

Learning to identify, respect and integrate hunger and satiety cues into one’s daily life are different steps of the IE implementation process. While most of the participants fell it was important to respect these physiological cues, dealing with them on a daily basis appeared to be challenging. With respect to hunger cues, the main barriers were confrontation with family obligations or schedules. Anticipating one’s need to eat and allowing oneself some flexibility in one’s eating rhythm were seen as facilitators. Barriers to respecting satiety cues were self-indulgence in food or the influence of others. Self-assertion and the creation of a pleasant meal environment were identified facilitators.

To the question “*What indicates to you when it’s time to eat?”*, five of six participants responded that it was generally their hunger cues. Some of the women tried to use this physiological signal to create a rhythm in their eating schedule. “*I’m not going to eat because it’s lunchtime. I’ll eat because I’m hungry. So sometimes I eat at three in the afternoon [when feeling hungry]*" (P5). But other women seemed to find it difficult to permit this flexibility in their daily lives. Several barriers to respecting hunger cues were described, such as having to follow family obligations or respect schedules. For P3, living with family members required adapting to them and putting her own needs aside, which resulted in eating without feeling hungry: “*because there was always the family priority*” (P3). Waiting to be hungry before a meal was therefore not always possible: "*that’s probably the whole difficulty, it’s not actually when I need to eat, it’s when the time allows me to eat, the break time, this is the schedule actually*” (P3). As mentioned by P5, eating when hungry required a great deal of flexibility. One participant imagined that living alone would have facilitated the process: “*If I was all alone, maybe there wouldn’t be any obligations with schedules either, it would be easier*” (P2). Anticipating and eating a small snack ahead of time, rather than waiting until she was too hungry, also helped P1 not to overeat during her next meal.

The participants were at different stages of the process of identifying satiation and respecting satiety cues. “*I mean, there are some who do it automatically, but for me, I had to learn to tell myself ‘yeah right, I need to stop [eating]'*" (P6). Barriers included eating too quickly, with the consequence of feeling satiety when it had already been exceeded. The facilitators used to identify satiety were to eat more slowly, to be more attentive to the process of satiation, and to create a pleasant meal environment. Planning enough time during meals also allowed some women to consciously taste the food and feel gustative satisfaction. Once satiety is identified, however, the challenge is to respect it. For three participants, self-indulgence in food and the desire for more food were barriers to respecting their satiety cues. For example, one woman spoke of a contradiction between her head and her stomach: “*My brain tells me ‘Stop, you’ve eaten enough’ and then I can’t stop. On the one hand it tells me ‘Stop, you’ve had enough’ and on the other hand it tells me ‘Eat some more'.*" (P1). Another woman (P3) could not “*control*” the urge to refill her plate. The influence of relatives and friends was also a barrier to respecting satiety. Two participants mentioned their identity within their family or a group of friends, which could be difficult to change. For example, if one of the women did not refill her plate as she had done previously, “*They [her friends] feel like I don’t like to eat at their house anymore*” (P6). Incentives to eat more, such as insisting on refilling a plate or tasting a dessert, could lead to oversatiation. In these situations, self-assertion was seen as a facilitator for respecting satiety.

## Theme 3: Making nutritious food choices for one’s body

A trend towards a nutritious and balanced diet emerged from the interviews. Trusting one’s body to guide food choices and finding satisfaction in the consumption of balanced and nutritious foods helped some participants to evolve through the IE implementation process. Using a balanced diet with the aim of losing weight, however, could be a barrier to IE.

Several of the interviewed women emphasised a high-quality, healthy and balanced diet. It was seen as important to follow the seasons, to eat less meat, to pay attention to fat consumption, and to the origin of food by favouring local products, to choose fresh products and to avoid pre-cooked meals. Several participants also stressed the importance of eating vegetables regularly. Finding gustative satisfaction in the consumption of foods that would contribute to a ‘*balanced diet’* facilitated these choices. “*The pleasure of eating something satisfying and balanced. That’s what’s important to me*” (P2). For one participant (P5), listening to and learning to trust her body helped her make food choices that were nutritious and suited her better. “*When I’m hungry, I have to eat. I don’t want to eat something sweet. I need a good meal, something warm […]*” (P5). As long as it was not seen as a constraint, making food choices for one’s body and one’s health therefore seemed to be a dimension that supported IE. On the other hand, following the rules of a balanced diet with the idea of controlling one’s weight goes against IE principles. As P3 noted, “*I conditioned myself to eat things that I didn’t want to eat*”. In doing so, she used to force herself to eat a vegetable soup in the evening to “*make it light*,” which induced food compensation out of frustration. After starting treatment at the Metabolic Centre, she realised the importance of seeking food satisfaction in her food choices rather than just eating healthily: *“if I’m happy with what I’ve eaten, it’s very rare that I will search for something to snack on later* " (P3).

## Theme 4: Struggling with emotional eating

Emotional eating was an issue for all participants, and many triggers were identified as barriers to the IE implementation process. The named triggers included upsetting events, mood fluctuations, or the need to overeat to inhibit emotions. Strategies to reduce emotional eating were self-assertion, welcoming emotions and taking care of them, engaging in pleasant activities, and perceiving emotionally charged situations differently.

All participants had experienced or were still struggling with emotional eating. “*I eat sometimes because of my emotions. Emotions take a lot of space in my life. And when I feel an emotion, I could eat anything”* (P5). One of the triggers of emotional eating was the need to overeat to inhibit emotions: “*for me, I’ve always said, it’s about filling me up. [*…*]. Because filling me up meant I was keeping the emotions down. I had food and it filled me up*” (P5). P2 spoke instead of the need to seek relief in food when difficult events occurred throughout her life. Upsetting events were also identified as a trigger for emotional eating in three women: “*sometimes when I’m upset, I tend to eat*” (P1). A bad mood or, on the contrary, a moment of well-being during a good meal, could trigger emotional eating. Yet some participants had developed strategies to reduce emotional eating, which represented facilitators of IE. One of these strategies, namely going to psychotherapy, helped three participants in the process. One woman (P3) described that she wanted to put “*the body and the mind in tune*” with psychological support. Another participant learned to welcome and take care of her emotions. “*My emotions, I have to take care of them. Because it’s not okay to always seek comfort in food”* (P5). Other strategies to deal with emotional eating were named, such as engaging in pleasant activities, perceiving emotionally charged situations differently, and developing self-assertion: *“Also, always adapting myself to others*… *in the end, they can also adapt to me from time to time!”* (P2).

## Theme 5: Learning to accept one’s body

Learning to accept one’s body was an important step in the IE implementation process, since many women seemed to suffer from body dissatisfaction and significant weight concerns. This combination led some of the participants to compensate with food or to diet, which represented a barrier to IE. Facilitators of body acceptance included rethinking weight loss expectations and taking care of one’s body, for example by practising physical activity for well-being rather than weight loss.

When asked about how they valued their physical appearance, five of the six women gave a rather negative description of their bodies. For many women, excess weight was a factor that negatively impacted their body acceptance. “*With this weight, I feel like I’m not such a pretty woman anymore” (P3).* Two participants have always felt dissatisfied with their body size. While most of the women were dissatisfied with their physical appearance, they did manage or at least tried to accept their body as it was, as illustrated by P3: *“So I don’t love myself as I am, that’s for sure. Now I’m not that hard on myself. It’s difficult at times, but in my everyday life I think that I accept myself quite well as I am”*. Learning to accept one’s body as it is then also meant accepting one’s body weight. For example, P2 thought that, realistically, she would never be thin again. This allowed her to let go of her unattainable weight loss expectations and accept her actual weight. However, some participants’ discourse about accepting their body weight was nuanced by lingering high expectations of weight loss, again reflecting unresolved ambivalence. Weight gain also became a real fear for two participants: “*I’m really nervous [about weight gain]. This fear becomes overwhelming.”* (P3). Gaining weight made them feel guilty, demoralised, and even devalued. One participant (P1) used food to compensate for her frustration at gaining two kilos. Conversely, another participant used to restrict her diet when she gained weight: “*Today I have to avoid this, I have to eat less*” (P4). These examples demonstrate the difficulty of moving away from weight loss diets when confronted with significant weight concerns and unresolved ambivalence. Body dissatisfaction marked by excessive weight preoccupation could therefore be a barrier to IE implementation.

The interviews revealed that ageing was accompanied by evolving viewpoints concerning body image and health concerns. One participant (P4) expressed a shift from being concerned about her physical appearance when she was younger to currently being mainly concerned about health problems related to her weight. Many participants expressed their motivation to lose weight in order to relieve joint pain, to improve their feeling of energy and agility, and to regain better breathing. This shift from physical appearance to health-related problems could encourage them to take care of their bodies, thus facilitating the acceptance of their body and of the IE approach. For example, one participant went through a period “*where you avoid looking at yourself and then you don’t even take care of your body anymore, not even using body lotion because you have dry skin, you don’t care because it’s useless anyway*.” (P2). Over time she came to think that she had to take care of her body anyway. Physical activity was described by three participants as a way of taking care of their bodies, as it helped them to feel more comfortable, to maintain mobility, or to achieve physical and mental well-being.

## Theme 6: Challenging weight stigma and sociocultural norms of beauty and thinness

Challenging weight stigma and sociocultural norms emerged as an essential step of the IE process. The confrontation with sociocultural norms of beauty and thinness, as well as the stigmatisation of people classified as ‘overweight’ or ‘obese’, could induce a desire to lose weight and to diet, hence representing a barrier to the IE implementation process. Aware of the omnipresence of these beauty ideals and the resulting weight stigma, some participants tried to challenge these norms and to detach themselves from their negative influence. Developing self-assertion, learning to listen to oneself and regaining self-confidence were identified as facilitators of this process.

Sociocultural norms of beauty and thinness were omnipresent throughout the interviews. For example, the ideals of beauty and thinness in the fashion world had a strong effect on the perception of women’s bodies. One participant, who worked in an underwear shop, regularly heard from her clients: " *they have too much belly, too much bottom, their breasts are too big, hips too large, shoulders too wide [*…*]. And although they are absolutely beautiful and have great bodies, they don’t feel good about themselves. And that’s typical of women, too*…*”* (P3). She added: “*in fact, my head is always thinking about body shapes, all the time*”. This had a strong effect on her functioning, and reinforced her own desire to lose weight. For her, the only way to achieve weight loss was to diet. This mirrored her ambivalence with, on the one hand, her criticism of these sociocultural norms of beauty and thinness, and, at the same time, her difficulty in detaching herself from them. The desire to lose weight in order to meet the sociocultural norms of thinness could activate dieting behaviour and was thus a barrier to the process of IE implementation. Weight stigmatisation was also part of the participants’ experiences: “*people see me through my weight, in fact, as if they don’t consider the qualities I have, the skills I have, but see me only as being overweight*” (P3). People classified as ‘overweight’ or ‘obese’ are also subjected to many unpleasant remarks linked to certain stereotypes: “*this person is large because she doesn’t stop eating*” (P2). Weight stigma may affect self-confidence and increase vulnerability to the dieting industry. Learning to listen to oneself and regaining self-confidence helped some women to challenge and to increase their detachment from these sociocultural standards of beauty and thinness. This facilitated learning to live in a world that stigmatises persons classified as ‘overweight’ or ‘obese’. “*I have to learn to listen to myself and not to others. That’s new for me.*” (P5). On the other hand, self-assertion was a learning experience for most of the participants. It enabled some of them to express themselves (P4) and to protect themselves from other people’s remarks and judgements.

## Discussion

This study explored how postmenopausal women classified as ‘overweight’ or ‘obese’ experienced the IE implementation process, and the barriers and facilitators that they encountered in their daily lives. The results of this qualitative research demonstrated a generally good acceptance of the IE approach by the participants, although its implementation was challenging. Compared to the many weight loss diets they had followed in their lives, the women saw IE as a radically different approach. The IE implementation process helped them to manage and integrate hunger and satiety cues into their daily lives, to discover the importance of food satisfaction, and to accept their bodies. Table 2 shows the overlap between the two complementary approaches of the 10 IE principles by Tribole and Resch (2012) and the three axes of the GROS (Sweerts et al., 2020) and it schematises the links between the themes that emerged from the thematic analysis.

The interviews highlighted the development of scepticism about weight loss diets (Theme 1). Interestingly, becoming sceptical about dieting after experiencing weight regain was part of the determinants of following non-dieting approaches in a qualitative study with 21 adults classified as ‘overweight’ or ‘obese’ (Leske et al., 2012). This could indeed have facilitated IE acceptance and its implementation among the participants of the present study. It is therefore advisable for therapists to guide their patients through a critical assessment of weight loss diets to increase this scepticism. This first theme was in line with the results of the qualitative study from New Zealand by Barraclough et al. (2019) on the barriers and facilitators of the IE learning process, where weight loss diets were experienced as sources of deprivation, leading to frustration and food compensation, and resulting in a strong sense of guilt. According to Tribole and Resch (2012), it is essential to start the IE implementation process by moving away from weight loss diets. The first IE principle is therefore to “*Reject the diet mentality*”, because the hope of finding a new and better weight loss diet may strengthen mental control of one’s diet, to the detriment of food-related cues (Tribole and Resch, 2012), and thus be a barrier to the IE implementation process. In France, the triaxial approach of the GROS also helps patients move from mind control to the physiological, sensory and intuitive regulation of one’s diet by working on a reduction of dietary restraint (Sweerts et al., 2020). Results regarding the association between dietary restraint and the likeliness of dieting or showing disinhibited eating behaviour (Quick and Byrd-Bredbenner, 2012) further demonstrate the importance of rejecting a diet mentality and reducing dietary restraint at the beginning of the IE implementation process.

Whereas some women in the present study had decided to move away from weight loss diets, the majority still showed some ambivalence about avoiding dieting behaviour because they were confronted with significant weight concerns. Even if they had experienced the undesirable consequences of weight loss diets, they struggled to move away from them, and still tried to restrict their diet in order to lose weight. This ambivalence was probably the result of several factors, including the internalisation of and comparison to unrealistic sociocultural ideals of thinness, which can lead women to develop body dissatisfaction and to desire to lose weight (Thompson and Stice, 2001; Van Den Berg et al., 2002). This drive for thinness has also been demonstrated in women aged 60 to 75, where body image concerns have been associated with dieting behaviour (Carrard et al., 2020). The presence of weight-related body dissatisfaction among some participants of the present study could therefore have encouraged them to control their diet with the goal of weight loss, thus reinforcing their dieting mentality, which is a significant barrier to IE. In addition, as Erhardt (2021) pointed out in her qualitative study on learning IE, it proves very challenging to accept one’s body in the Western culture of dieting and slimming. Thus, adhering to the IE approach seems to require a constant effort to resist the dominant dieting culture in Western society (Erhardt, 2021). An essential step in IE implementation was therefore to learn to accept one’s body (Theme 5) and to work on weight loss goals and expectations. This process converges with Principle Eight of Tribole and Resch (2012), “*Respect your body*”. According to this principle, accepting one’s body morphology and weight is a critical step because as long as someone struggles with their body acceptance, it is difficult to develop a peaceful relationship with food. In line with this assumption, a pilot study evaluated the effect of an online prevention program promoting intuitive eating and body satisfaction among 323 women aged between 18 and 75 years. The results showed a significant and stable reduction in restrictive eating and shape and weight concerns (Beintner et al., 2019). Taking into account that body dissatisfaction persists with age (Cameron et al., 2019), these findings further underline the importance of integrating aspects of body image into the IE implementation process of postmenopausal women.

The experienced ambivalence could also be influenced by the internalisation of health standards encouraging participants to reach a “healthy weight” (Rodgers, 2016). According to Rodgers' (2016) “healthy weight sociocultural model”, the “healthy weight” discourse increases the belief that weight is mainly controllable through diet and physical activity, thus increasing personal responsibility for being classified as ‘overweight’ or ‘obese’. This may encourage dieting if one’s weight does not meet the standardised weight criteria. As a result, incentives to reach a “healthy weight” range cause tension with IE anti-diet principles. Clinicians should therefore be careful not to use a weight-centred health discourse when accompanying patients through the IE implementation process, but instead, focus on a discourse improving health and well-being.

As part of the IE process, the participants were learning how to identify and respect their hunger and satiety cues, and how to integrate these physiological signals into their daily lives (Theme 2). This learning process is part of the first axis of the GROS (Sweerts et al., 2020) as well as the “*Honour your hunger*” and “*Feel your fullness*” principles of Tribole and Resch (2012). Nevertheless, the women encountered several societal barriers, including family obligations and schedules to follow, as well as the influence of others, which led many of them to eat without hunger or to exceed their satiety. Barraclough et al. (2019) also highlighted the strong relationship between food and social interaction in their study, which was perceived as a barrier by many of their participants. As a result, for most of the women of the present study, it was essential to give due importance to food cues, even though respecting them proved to be difficult in everyday life. Some facilitators, however, were identified to help in dealing with food-related cues. Some women advocated allowing themselves flexibility in their eating rhythm. However, in a daily life where flexibility is difficult to achieve, it might be advisable to rely on the anticipation of needs to facilitate food management. Zermati (2011), a nutritionist doctor and co-founder of the GROS association, calls this “*predictive appetite*”, which allows eaters to adapt the frequency and size of their meals according to their social constraints. Tribole and Resch (2012) recommend following “*practical hunger*”, which means remaining practical about one’s eating rhythm and anticipating times when it is not possible to eat. In these cases, it would be better to eat a small snack when the opportunity arises, even if hunger is low, rather than to wait for hunger to intensify and then overeat at the next meal (Tribole and Resch, 2012). In practice, learning to deal with hunger and satiety cues is a gradual process. These signals need first to be experienced and then integrated into one’s daily life. Afterwards, one can learn to trust the cues to guide food choices and the quantities to eat at each meal.

These experiences are the basis on which gentle nutrition can be learned. According to the last principle of Tribole and Resch (2012), “*Honour your health with gentle nutrition*”, gentle nutrition advocates making nutritious food choices not for weight loss, but to improve health as a whole. A high-quality and balanced diet was of great importance for several participants in this study (Theme 3). It is interesting to note that the region in which the interviewed women lived is an important agricultural area (Department of Finance and External Relations, 2018; Statistical Office, 2013). It is possible that this characteristic affected the participants’ consumption of fresh and local products, and may have facilitated their adoption of gentle nutrition. However, making nutritious food choices was not yet a guarantee of gentle nutrition as defined by Tribole and Resch (2012), because until one achieves a healthy and peaceful relationship with one’s diet, it might be difficult to eat a balanced diet without being on a new weight loss diet (Tribole and Resch, 2012). Whereas this last IE concept was difficult to implement for several women struggling to move away from the diet mentality, two of the participants succeeded in doing so by listening to their bodies, respecting their food-related cues, developing food satisfaction, and allowing themselves to eat all types of food without feeling guilty. This is in line with the results of Barraclough et al. (2019), where learning to trust one’s body to guide food choices and the quantities to eat were facilitators of this last principle. Combining intuitive eating and nutrition knowledge, however, may be seen as a challenge. Bearing this in mind, it is important to be cautious as a clinician when discussing recommendations for healthy and balanced diets with patients, so as not to increase diet mentality.

Several participants explained that they had used or were still using food as a way to cope with emotions (Theme 4). This is not surprising, given that a large cross-sectional study in France (Péneau et al., 2013) found a high prevalence of emotional eating among people classified as ‘overweight’, especially among women. In the same study, emotional eating was associated with dieting, leading the authors to question the tendency to overeat in response to emotions among persons following weight loss diets (Péneau et al., 2013). This association again highlights the importance of moving away from weight loss diets in order to go through the IE implementation process, as proposed by the chronology of Tribole and Resch (2012). On the other hand, emotional eating was identified as a barrier to IE implementation, which is in line with the results of Barraclough et al.’s (2019) qualitative study. According to the seventh principle of Tribole and Resch (2012) “*Cope with your emotions without using food”*, seeking distraction or inhibiting one’s emotions through food may lead to a disconnection from one’s hunger and satiety cues. In any case, identifying the triggers of emotional eating and accepting one’s emotions are the first steps to reducing emotional eating. This is addressed in the emotional axis of the GROS (Sweerts et al., 2020).

Self-assertion was identified as a facilitator of IE implementation at different levels in the interviews. Whether it was to challenge the sociocultural norms of beauty and thinness (Theme 6), to assert oneself in front of others in order to resist their influence, or to assert oneself against the dietary beliefs conveyed by numerous weight loss diets, self-assertion allowed the participants to develop scepticism about weight loss diets and even for some participants to move away from dieting behaviour, to trust their hunger and satiety cues, and to accept their physical appearance. It also helped some women to gain confidence in a weight-stigmatising world (Theme 6). This is especially important because women classified as ‘obese’ are shown as more likely to perceive discrimination related to their weight (Spahlholz et al., 2016). Weight stigma has also been associated with body image dissatisfaction, weight concerns and binge eating (Annis et al., 2004), making weight stigmatisation a potential barrier to IE. The third axis of the GROS “*self-acceptance*”, addresses the development of self-assertion, self-love and self-respect in a weight-stigmatising world (Sweerts et al., 2020), thus allowing a link with the last theme of this study (Theme 6). In line with this concept, increased self-compassion was reported as a result of IE implementation in a pilot study (Burnette et al., 2022). Although self-acceptance is not explicitly described in the 10 IE principles of Tribole and Resch (2012), it deserves to be integrated into the treatment of patients suffering from weight stigma, low self-esteem, or the social pressures of thinness, in order to help them challenge their weight ideals, reduce weight concerns and resist the temptations of the numerous weight loss diets, and thus facilitate the IE implementation process.

In summary, this qualitative study among postmenopausal women confirmed the presence of a preoccupation with weight, emphasised by the confrontation with and internalisation of sociocultural standards of beauty and thinness and weight stigmatisation. Significant weight concerns, which led to ambivalence about the desire to move away from weight loss diets, were thus a hindrance to the IE implementation process. However, as experience grows with age, the accumulation of the negative impacts of dieting in this sample might have favoured the development of scepticism towards weight loss diets and facilitated IE implementation. Although respecting food cues has gained importance among the participants, their integration into everyday life seemed difficult when confronted with family obligations, schedules, or the influence of others. Allowing oneself flexibility in the daily eating rhythm and anticipating one’s needs therefore facilitated IE implementation. If emotional eating was a barrier to IE, identifying and accepting one’s emotions was a necessary step in the implementation of IE. Furthermore, developing food satisfaction for nutritious foods helped some participants to engage in gentle nutrition. Finally, developing self-assertion was identified as a facilitator at different levels of IE implementation.

The present research complements the small number of qualitative studies on IE, and to our knowledge, provides, the first insight into the experience of implementing IE with postmenopausal women. The novelty of this research is that it brings into focus the barriers and facilitators of this process by combining two highly complementary approaches of IE, notably the triaxial approach of the GROS and the 10 IE principles of Tribole and Resch (2012). Although a certain chronology in implementing IE makes sense, as proposed by Tribole and Resch (2012), the themes identified in the present study demonstrate the relatively iterative nature of the IE implementation process. It therefore seems important to adapt the process to the initial situation and evolution of each patient, and to return to a theme if it needs further exploration. IE encompasses not only hunger and satiety cues, but also emotional eating, body image and physical activity, gentle nutrition, and self-assertion. In order to facilitate the implementation of IE, it is therefore essential to include the expertise of different health professionals, such as psychologists, experts in adapted physical activity, and dieticians. Knowledge of the barriers and facilitators of the IE implementation process may also guide clinicians in helping patients overcome their difficulties and improve their compliance with this anti-diet approach.

### Limitations, strengths and suggestions for further research

This qualitative research provided a rich description of the barriers and facilitators experienced in the IE implementation process of postmenopausal women classified as ‘overweight’ or ‘obese’ from a rural area in French-speaking Switzerland. The transferability of these results therefore applies to this population, and claims made based on this sample may not apply to other individuals experiencing IE. Even though the recruitment of six participants was enough to carry out a thematic analysis according to Braun and Clarke (2013), the small number of participants in this study did not allow for data saturation (Bradshaw et al., 2017). In addition, the framework of this study did not allow data triangulation from other researchers nor member-checking by the participants to verify the accuracy of the transcripts (Lincoln and Guba, 1985). This means that some subjects might have been undermined. No IE implementation protocol was pre-established at the Metabolic Centre, which may explain the somewhat different therapeutic approaches from one therapist to another, and may have influenced the participants’ experience of IE implementation. In order to limit social desirability bias (Butori and Parguel, 2012), only patients who had not been treated by the first author were recruited. Nevertheless, a risk of bias must be considered, as the participants were aware of the interviewer’s role as a dietician within the Metabolic Centre. For example, this might have encouraged participants to overrate the importance of eating fruits and vegetables.

It seems important to identify the role played by the preconceptions and values of the dietician who conducted the interviews. Having seen the potential side effects of restrictive eating and dieting in many clients, she was convinced of the benefits that IE had on psychological well-being and health factors. These preconceptions may have affected the construction of this research, the way the interviews were conducted and the interpretation of the results. This subjective character is nevertheless valued in a subjectivist research context. In order to reduce these influences, the first author followed a process of reflexivity. She confronted her preconceptions with the scientific literature, acquiring a critical perspective on the subject. Discussions with the co-author helped her to distance herself from her position as a clinician. In the end, this enabled the first author to develop a more objective conception of IE and to open her perspectives on the difficulties that could be encountered in implementing IE.

More experimental and longitudinal studies are needed to confirm the effects of IE implementation on eating disorders, body appreciation and health indicators, particularly in postmenopausal women. Although the number of cross-sectional studies on IE is increasing, there is still little data on which to base implementation. IE studies tend to incorporate a wide variety of elements into their interventions, making it difficult to isolate a single effective component (Warren et al., 2017). It would therefore be useful to identify the components of IE implementation interventions that are effective rather than their overall approaches. It is also essential to determine which patients could most benefit from these interventions. It is important to gather more knowledge about the acceptability of IE and the factors affecting its implementation. To do so, future qualitative studies should recruit a larger number of participants from different samples, based for example on age, gender or body size.

## Conclusion

During the process of IE implementation, the women interviewed in this qualitative study have developed significant scepticism about dieting, which in turn reinforced their desire to move away from weight loss diets. A major obstacle remained, however, in the numerous incentives for weight loss diets present in Western society, which are exacerbated by the thinness ideal and the stigmatisation of postmenopausal women being classified as ‘overweight’ or ‘obese’. The resulting body dissatisfaction and weight concerns reinforced the women's intentions of weight loss, creating an ambivalence among the participants towards IE. Learning to accept one’s body and developing self-assertion by challenging weight stigma and the sociocultural norms of beauty and thinness can facilitate the implementation of IE. Most women developed a more peaceful relationship with their diet through this process. The IE approach, although challenging to implement, was therefore accepted as a most welcome alternative to dieting.

## Note

1. According to the WHO classification, a BMI less than 18.5 kg/m^2^ falls within the ‘underweight’ range, a BMI between 18.5 and 24.9 kg/m^2^ falls within the ‘healthy’ weight range, a BMI between 25.0 and 29.9 kg/m^2^ falls within the ‘overweight’ range and a BMI equal or higher than 30.0 kg/m^2^ falls within the ‘obesity’ range. BMI should not be used to diagnose body fat mass or health. We acknowledge that the use of these terms may reinforce the stigma surrounding weight and the “healthy weight discourse” pointed out by Rodgers' sociocultural model (2016). ‘Overweight’ and ‘obesity’ are denominations still widely used in hospitals and specialized services, and we use them in this article at a descriptive level.

## Appendix

### Annex 1 – interview guide

Opening question: How long have you been undergoing treatment at the Metabolic Centre?

Questions related to the eating behaviour:

1. How do you decide when to eat? And when to stop eating?
2. How much do you trust your body to tell you when, what and how much to eat?
3. What do you pay attention to when making food choices? What is important to you?
4. Are there any foods that you avoid eating? If so, which ones and why? How do you feel when you eat these foods?
5. Do you follow dietary rules that help you?
6. Do you ever eat to relieve your emotions (e.g. boredom, sadness, anger, frustration, stress, anxiety…)? If so, how do you do it?
7. What do your relatives think of your eating habits? What effect does this have on the way you eat? What support do you get from your relatives in managing your eating behaviour and/or eating habits?
8. Have your eating habits and the way you eat changed in recent years? If so, how and for what reasons?
9. What do you think helps you to manage your eating behaviour (specify)? And what makes it difficult to manage your eating behaviour (specify)?
10. What would you need to better manage your eating behaviour (specify)?

Questions related to the body image:

1. Are you concerned about your body weight?
  1.1. What are the most important reasons for you to manage your weight?
1.2. Tell me what happens when you see or feel that your weight is increasing/decreasing? How does this affect your life (mood, body satisfaction, diet)?
2. Are you concerned about your physical appearance? If so, why?
  2.1. What do you think affects your body image (media, relatives, friends, society …)?
  2.2. What do you do in order to create a physical appearance that you like?
  2.3. What do you do in order to take care of your body?
2.4. What do others think of your physical appearance (comments from family, friends, colleagues)? What effect does this have on you (positive or negative)? How much does this affect the way you perceive yourself?
3. How have concerns about your body weight and/or body image evolved in recent years?
  3.1. How much has this affected the way you eat?
4. Would you like to add/clarify anything in this interview?

#### Sociodemographic questions:

- What is your marital status?
- Do you have children? If so, how many and how old are they?
- What is your professional status?
- What is your educational background?
- What is your current weight? your current height?
